# Characterizing Brain Iron Deposition in Patients with Subcortical Vascular Mild Cognitive Impairment Using Quantitative Susceptibility Mapping: A Potential Biomarker

**DOI:** 10.3389/fnagi.2017.00081

**Published:** 2017-03-30

**Authors:** Yawen Sun, Xin Ge, Xu Han, Wenwei Cao, Yao Wang, Weina Ding, Mengqiu Cao, Yong Zhang, Qun Xu, Yan Zhou, Jianrong Xu

**Affiliations:** ^1^Department of Radiology, Ren Ji Hospital, School of Medicine, Shanghai Jiao Tong UniversityShanghai, China; ^2^Department of Neurology, Ren Ji Hospital, School of Medicine, Shanghai Jiao Tong UniversityShanghai, China; ^3^GE Applied Science Laboratory, GE HealthcareShanghai, China

**Keywords:** subcortical ischemic vascular disease, subcortical vascular mild cognitive impairment, quantitative susceptibility mapping, iron deposition, gray matter nuclei

## Abstract

The presence and pattern of iron accumulation in subcortical vascular mild cognitive impairment (svMCI) and their effects on cognition have rarely been investigated. We aimed to examine brain iron deposition in svMCI subjects using quantitative susceptibility mapping (QSM). Moreover, we aimed to investigate the correlation between brain iron deposition and the severity of cognitive impairment as indicated by *z*-scores. We recruited 20 subcortical ischemic vascular disease (SIVD) patients who fulfilled the criteria for svMCI. The control group comprised 19 SIVD patients without cognitive impairment. The SIVD and control groups were matched based on age, gender, and years of education. Both groups underwent QSM using a 3.0T MRI system. Susceptibility maps were reconstructed from *in vivo* data, which were acquired with a three-dimensional spoiled gradient recalled sequence. Then, regions of interest were drawn manually on the map of each subject. The inter-group differences of susceptibility values were explored in deep gray matter nuclei, including the bilateral pulvinar nucleus of the thalamus, head of caudate nucleus, globus pallidus, putamen, hippocampus, substantia nigra, and red nucleus. The correlations between regional iron deposition and composite *z*-score, memory *z*-score, language *z*-score, attention-executive *z*-score and visuospatial *z*-score were assessed using partial correlation analysis, with patient age and gender as covariates. Compared with the control, the svMCI group had elevated susceptibility values within the bilateral hippocampus and right putamen. Furthermore, the susceptibility value in the right hippocampus was negatively correlated with memory *z*-score and positively correlated with language *z*-score. The susceptibility value in the right putamen was negatively correlated with attention-executive *z*-score in the svMCI group. However, composite *z*-score were unrelated to susceptibility values. Our results suggest that brain iron deposition has clinical relevance as a biomarker for cognition. In addition, our results highlight the importance of iron deposition in understanding svMCI-associated cognitive deficits in addition to conventional MRI markers.

## Introduction

Vascular dementia (VaD) is clinically characterized by stepwise progression, fluctuating course, and predominant deterioration of intelligence with the relative preservation of personality. VaD is the second most common form of dementia after Alzheimer’s disease (AD) and places an enormous burden on society (Erkinjuntti, 1999). In China, it is estimated that the crude incidence in persons ≥65 years was 3.1/1000 person-years for VaD (Yuan et al., 2016). Subcortical vascular dementia (SVaD), a small vessel disease (SVD), constitutes approximately half of VaD cases (Yoshitake et al., 1995). Recently, most studies have involved patients in the prodromal stage of AD, referred to as the amnestic mild cognitive impairment (aMCI). It is also clinically important to focus on subcortical vascular mild cognitive impairment (svMCI), which is a prodromal stage of SVaD and distinctive from aMCI (Lee et al., 2014), since management of risk factors and drug treatment could prevent the evolution of svMCI to SVaD (Seo et al., 2010). Finding potential biomarkers for early diagnosis and relating these biomarkers to cognitive measurements before the onset of clinical deterioration are urgent matters.

Patients with svMCI have been shown to exhibit cognitive impairments in executive, language, visuospatial, and memory functions (Seo et al., 2009, 2010) and have been associated with structural and functional alterations in widespread regions (Seo et al., 2010; Yi et al., 2012, 2015; Yoon et al., 2013; Kim et al., 2014), while these functions are further impaired in cases of SVaD (Kim et al., 2014). In svMCI patients, subcortical areas, such as the basal ganglia and thalamus, are more prominently involved than the frontal region. Functional changes in the very early stages of svMCI or SVaD likely begin in subcortical structures and may progress to the frontal cortices in the later stages of the disease (Seo et al., 2009). Structurally, specific pattern in hippocampal atrophy progressed has been reported in patients with svMCI, exhibiting focal atrophy in the lateral body, while additional atrophy in the lateral head and inferior body in SVaD patients (Kim et al., 2014). The region-specific vulnerability of hippocampal subfields to svMCI pathology has also been observed in the left subiculum/presubiculum and in the right cornu ammonis4/dentate gyrus (Li et al., 2016). Previous PET studies showed that hypometabolism in the deep structures and frontal region of the brain is a sensitive marker for SVaD. It could reflect disconnection from the basal ganglia/thalamus as well as among cortical regions, according to the concept of diastasis (Kerrouche et al., 2006). A susceptibility weighted imaging (SWI) study demonstrated that the severity of cognitive impairment is closely correlated with widespread abnormal iron deposition in the hippocampus, caudate nucleus, putamen, globus pallidus, substantia nigra, hippocampus, and caudate nucleus of SVaD patients (Liu et al., 2015a). These results implicate deep-structure areas in the pathophysiological mechanism of the evolution of svMCI to SVaD.

With the development of quantitative MRI techniques, assessment of iron levels has become more accurate and sensitive. Although SWI can takes advantage of the magnetic property properties to of create useful image contrasts, but it does not provide quantitative measures of magnetic susceptibility. This limitation addressed by quantitative susceptibility mapping (QSM), an MR technique that depicts and quantifies magnetic susceptibility sources. QSM computes the underlying susceptibility of each voxel as a scalar quantity (Liu et al., 2015b). The voxel intensity reflects tissue susceptibility to enable the quantitative investigation of iron concentration in the brain regions where iron is the dominant source of magnetic susceptibility (Bilgic et al., 2012; Langkammer et al., 2012; Li et al., 2015a). In addition, the phase or T2^∗^ contrast a weighted summation of the magnetic properties of the surrounding tissue, reflects only the “shadow” of the surrounding susceptibility sources. QSM can provide an accurate definition of the distribution of magnetic biomaterials in MRI through deconvolution (Wang and Liu, 2015). More importantly, magnetic susceptibility is a direct reflection of the molecular composition and cellular architecture of the tissue. Consequently, QSM is becoming a quantitative imaging approach for characterizing normal and pathological tissue properties by quantifying magnetic susceptibility (Liu et al., 2015c). Therefore, QSM is being evaluated in a growing number of clinical applications, including: (1) the separation of diamagnetic calcium from paramagnetic iron; (2) the quantification of myelination in the white matter; and (3) the quantification of iron deposition and blood by-products (Liu et al., 2015c). In recent studies, QSM has become increasingly prominent in the search for a quantitative biomarker for assessing the iron deposition. The spread of accumulated iron accumulation in the brain spreading across the cortex, cerebellum, and deep-brain nuclei was is age-correlated and occurs throughout the adult lifespan (Acosta-Cabronero et al., 2016). This is consistent with decay in the course of normal brain aging. Therefore, *in vivo* QSM is a useful non-invasive tool for investigating cerebral iron accumulation. Rapid iron accumulation in subcortical and deep structures is also an important developmental processes that contribute to cognitive functions (Darki et al., 2016). Thus, QSM can be used in future studies of predictive value for cognitive performance. Additionally, movement and neurodegenerative disorders, such as Parkinson’s disease (PD), VaD, and AD, are associated with iron overload in the brain (Acosta-Cabronero et al., 2013; He et al., 2015; Moon et al., 2016). Although it is unclear whether iron accumulation is the cause or consequence of these diseases, monitoring the spatial distribution and the temporal dynamics of iron deposition may offer important insights on the pathogenesis of these diseases. QSM can explain results in molecular terms and identify elevated iron levels to assist early disease diagnosis (Acosta-Cabronero et al., 2013; He et al., 2015). It has been proved that SVaD contributes to the process of increased iron accumulation in a SWI study (Liu et al., 2015a). Thus, iron accumulation should also be detected in svMCI. However, it is unknown whether brain iron concentrations in svMCI patients distinctly change compared with those in controls without cognitive impairment.

## Materials and Methods

### Subjects

Thirty-nine subcortical ischemic vascular disease (SIVD) subjects were recruited from patients who were admitted to the Neurology Department of Ren Ji Hospital from February 2015 to December 2015. Patients were evaluated by clinical interview, neurologic and neuropsychological tests, and brain MRI. In accordance with the criteria suggested by Galluzzi et al. (2005), SIVD was defined as subcortical white matter hyperintensities (WMHs) that are visible on T2-weighted imaging with at least one lacunar infarct. The exclusion criteria (**Table 1**) were applied as previously described (Sun et al., 2011, 2016). Patients were excluded if they presented with calcification or microbleeds in deep gray matter nuclei, including the bilateral pulvinar nucleus of the thalamus, head of caudate nucleus, globus pallidus, putamen, hippocampus, substantia nigra, and red nucleus with dark spots and negative susceptibility. Twenty patients with SIVD fulfilled the svMCI criteria suggested by Petersen et al. (1999) and by a recent study (Lee et al., 2014). The inclusion criteria are presented in **Table 1**. The control group comprised 19 SIVD patients with neuropsychological test scores within the normal range. Members of the control group were matched based on age, gender composition, and years in education. All the patients were right-handed.

**Table 1:** Exclusion criteria for SIVD and inclusion criteria for svMCI.

Exclusion criteria for SIVD
• Cortical and/or cortico–subcortical non-lacunar infarcts
• Watershed infarcts
• White-matter lesions of specific causes
• Neurodegenerative diseases (e.g., Alzheimer’s disease and Parkinson’s disease)
• Intracerebral hemorrhages
• Normal-pressure hydrocephalus
• Alcoholic encephalopathy or illicit drug use
• Patients with major depression (Hamilton Depression Rating Scale [HDRS] ≥ 18 (Hamilton, 1960), other psychiatric comorbidities or severe cognitive impairments
• MRI safety contraindications and claustrophobia
• Education < 6 years

**Inclusion criteria for svMCI**

• Subjective cognitive complaints reported by the participant or caregiver
• Basically normal activities of daily living (ADL)
• Quantifiable cognitive impairment within 1 or more domains (memory, attention-executive function, language or visuospatial function)
• No dementia

*SIVD, subcortical ischemic vascular disease; svMCI, subcortical vascular mild cognitive impairment.*

The objective of the present study is to investigate iron deposition in the deep gray matter nuclei of svMCI patients and its correlation with the severity of cognitive impairment. We utilize QSM to achieve this objective in the hopes of establishing a promising neuroimaging biomarker for the early diagnosis of svMCI.

The current study was approved by the Research Ethics Committee of the Ren Ji Hospital, School of Medicine, Shanghai Jiao Tong University. Written informed consent was obtained from each subject before participation. All procedures were in accordance with the institutional guidelines.

### Neuropsychological Tests

Neuropsychological assessments were completed by two experienced neurologists (QX and WC) within 1 week after MRI examination. No patients suffered transient ischemic attack or a stroke between the MRI examination and the assessment. All patients underwent a comprehensive battery of neuropsychological tests, including tests of all cognitive domains. The scales listed in **Table 2** were used as described in a previous study (Hachinski et al., 2006; Xu et al., 2014). The neurological features of each patient in the svMCI group are presented in **Table 3**.

**Table 2:** Comprehensive battery of neuropsychological tests used to evaluate cognitive status.

Cognitive function	Tests
Attention-executive function	Chinese modified version of the Trail Making Test (TMT)
	Modified version of the Stroop Color-Word Test (SCWT)
	Category Verbal Fluency Test (VFT)
Memory function	Chinese version of the Auditory Verbal Learning Test (AVLT) for short-delay and long-delay free recall
	Rey–Osterrieth Complex Figure (ROCF) delayed recall test (Chinese version)
Language function	Boson Naming Test (the 30-item version) to evaluate
Visuospatial function	ROCF copy test

**Table 3:** The neurological features of each individual svMCI patient.

	Att-exe	Memory	Language	Visuospatial
svMCI01	-	+	-	-
svMCI02	-	+	-	-
svMCI03	-	+	-	-
svMCI04	-	+	-	-
svMCI05	-	+	-	-
svMCI06	+	+	-	-
svMCI07	+	+	-	-
svMCI08	+	+	-	-
svMCI09	+	+	+	-
svMCI10	-	-	+	-
svMCI11	+	+	+	-
svMCI12	+	+	-	-
svMCI13	-	+	-	-
svMCI14	-	+	-	-
svMCI15	+	+	-	-
svMCI16	-	+	-	-
svMCI17	-	+	+	-
svMCI18	+	+	+	-
svMCI19	-	+	-	-
svMCI20	+	-	-	-

*svMCI, subcortical vascular mild cognitive impairment; Att-exe, attention-executive function; Memory, memory function; Language, language function; Visuospatial, visuospatial function.*

The *z*-score is a standard score. Raw scores are transformed into standard scores to facilitate interpretation. A *z*-score has a mean of zero and a standard deviation of one (Iverson, 2011). It provides a simple measure for the comparison of neuropsychological measures in terms of their deviation from the mean. In this study, the raw scores of each neuropsychological test were processed with z-transform. The *z*-scores of the respective test were averaged. Then, composite *z*-score were computed by averaging the *z*-scores of individual cognitive domains.

### MRI Data Acquisition

MRI scanning was conducted using a 3.0T MR system (Signa HDxt; GE HealthCare, Milwaukee, WI, USA) equipped with an eight-channel phased array head coil at Ren Ji Hospital. Foam padding was used to restrict the head motion of each patient. Earplugs were provided to reduce scanner noise. Phase images with whole-brain coverage were acquired using a standard flow-compensated three-dimensional spoiled gradient recalled (3D-SPGR) sequence with the following parameters: TE_1_/ΔTE/TE_16_ = 3.2/2.42/39.5 ms, TR = 42.5 ms, FA = 12°, bandwidth = 62.5 kHz, FOV = 220 mm × 220 mm, matrix = 256 × 256, slices = 66. This protocol resulted in an isotropic in-plane resolution (0.86 mm × 0.86 mm) with a slice thickness of 2 mm. The total acquisition time was about 4 min 27 s.

In addition to QSM images, following acquisitions were also performed to confirm the other absence of structural lesions: (1) 3D-SPGR sequence images (TR = 6.1 ms, TE = 2.8 ms, TI = 450 ms, FA = 15°, slice thickness = 1.0 mm, gap = 0, FOV = 256 mm × 256 mm, and slices = 166); (2) T2-fluid attenuated inversion recovery sequence (TE = 150 ms, TR = 9075 ms, TI = 2250 ms, FOV = 256 mm × 256 mm, and slices = 66); (3) axial T2-weighted fast spin-echo sequences (TR = 3013 ms, TE = 80 ms, FOV = 256 mm × 256 mm, and slices = 34).

### Image Reconstruction

**FIGURE 1:**
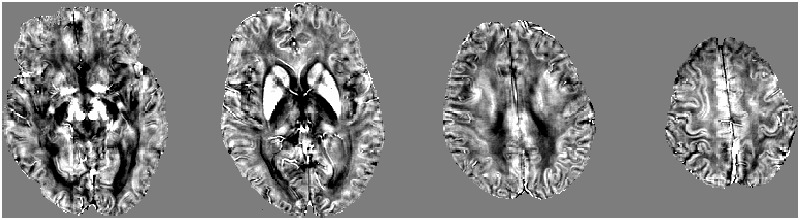
**A typical quantitative susceptibility maps with a 58-year-old subcortical vascular mild cognitive impairment subject**.

Before QSM reconstruction, the quality of the magnitude raw data was checked by two trained observers. The images were computed using a MATLAB-based software, called “STI Suite” (Li et al., 2014). The tool freely available at http://people.duke.edu/~tildecl160/ for non-commercial academic use. The method was performed as suggested in previous studies (Li et al., 2011; He et al., 2015). First, the image was reconstructed with 3D Fast Fourier transform using complex k-space data for each of the eight receiver coils and then separated into the magnitude and phase images. The magnitude image was obtained from the squared summation of the eight magnitude images. It was used for the extraction of the brain tissue. The Fourier transform of phase image for each individual coil was calculated from the original wrapped phase data. Then, the resultant phase Fourier transforms were averaged to yield the Fourier transform of the final combined signal phase. In the next step, the background phases were removed using the spherical mean value method with a filter radius of 8 pixels. All voxels contained in the sphere must be valid, which cannot be met at brain boundaries. Therefore, the filter radius was gradually reduced to the largest size possible at brain boundaries. Since the sphere size is finite, the phase generated by the brain tissue outside the sphere is harmonic in the sphere, and thus can also be removed along with the background phase. This removed low frequency phase from the brain tissue can be restored with a deconvolution operation (Li et al., 2011; Schweser et al., 2011). The frequency map was calculated from the resultant local phase image. In the final step, quantitative susceptibility map was calculated from the frequency map using an improved least-squares (iLSQR) method (Li et al., 2015b). The regularization threshold for Laplace filtering was set at 0.04 (He et al., 2015). **Figure 1** shows the reconstructed QSM images from a 58-year-old svMCI subject.

### Region of Interest Analysis

**FIGURE 2:**
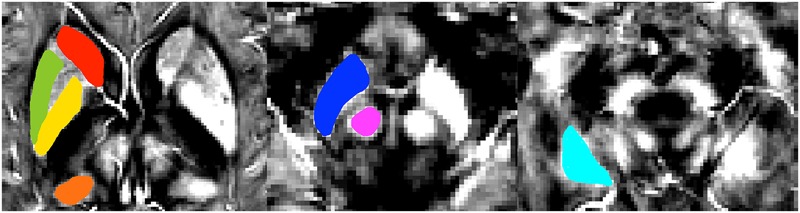
**A typical susceptibility map from one single patient with svMCI illustrates the regions of interest.** Colors represent areas of the brain: red = head of caudate nucleus; green = putamen; yellow = globus pallidus; orange = pulvinar nucleus of the thalamus; dark blue = substantia nigra; pink = red nucleus; light blue = hippocampus.

The QSM method has been successfully applied to detect the susceptibility values directly used for comparison without reference to any selected structures (Li et al., 2015a). Then, we selected iron-rich subcortical nuclei as regions of interest (ROI) to assess the association between the susceptibility values of these ROIs with cognitive performance. ROIs were selected in accordance with previous studies and included the pulvinar nucleus of the thalamus, head of caudate nucleus, globus pallidus, putamen, hippocampus, substantia nigra, and red nucleus (Liu et al., 2015a; Moon et al., 2016). **Figure 2** presents examples of the seven ROIs. The ROI were drawn manually on the susceptibility maps using MRIcro software available at http://www.mccauslandcenter.sc.edu/crnl/tools. Two trained neuroradiologists (YS and YW), who were blinded to the clinical data of the subjects, independently analyzed the MRI data.

### Statistical Analyses

All statistical analyses were performed using SPSS (v. 17.0; SPSS Inc., Chicago, IL, USA). To determine intergroup differences, the age, education, and *z*-scores of the svMCI and control groups were compared using an independent two-sample *t*-test. Gender heterogeneity between groups was assessed using the Chi-square test. Susceptibility values of the svMCI and control groups were compared using two-sample *t*-tests. For data that were not normally distributed, continuous variables were compared using the Mann–Whitney *U* test. The significance levels were set at *p* < 0.05 for all analyses. To assess the reliability of measurement in segmenting ROI, we selected the MR images of 10 normal subjects in accordance with a previous study (Moon et al., 2016). Another rater independently measured the ROIs for each anatomical target. Inter-rater reliability among all regions was 0.947 (95% confidence interval: 0.927–0.962, *p* < 0.001).

To identify the brain iron deposition in svMCI subjects correlating with the severity of cognitive impairment according to the *z*-score, the correlations the susceptibility values of each brain region showed group deference and the composite *z*-score, memory *z*-score, language *z*-score, attention-executive *z*-score and visuospatial *z*-score were assessed partial using correlation analysis, with patient age and gender as covariates (Persson et al., 2015).

## Results

### Demographics, Neuropsychological Scores, and MRI Data Analysis

Demographic characteristics and main neuropsychological information are shown in **Table 4**. No significant differences in age, gender, and education were found between the two groups. The svMCI group had significantly lower composite *z*-score, attention-executive *z*-score, memory *z*-score and language *z*-score. The svMCI patients and control groups displayed no difference in terms of visuospatial *z*-score.

**Table 4:** Demographic, *z*-sores for svMCI group and control.

	svMCI (*n* = 20)	Control (*n* = 19)	*p*-value
Age (year)	63.40 ± 7.98	65.11 ± 3.71	0.396
Gender (male/female)	13/3	15/4	0.622
Education(year)	10.70 ± 3.48	12.00 ± 2.56	0.194
Composite	-0.50 ± 0.51	0.30 ± 0.45	**<0.001**
Att-exe	-0.79 ± 0.82	0.18 ± 0.48	**<0.001**
Memory	-1.69 ± 0.54	-0.21 ± 0.76	**<0.001**
Language	-0.59 ± 1.31	0.38 ± 0.90	**0.011**
Visuospatial	1.07 ± 0.45	0.86 ± 0.87	0.365

*svMCI, subcortical vascular mild cognitive impairment; Composite, composite *z*-score; Att-exe, attention-executive *z*-score; Memory, memory *z*-score; Language, language *z*-score; Visuospatial, visuospatial *z*-score.*

*Dates are given in means ± standard deviations; *p*-value < 0.05 was considered to be statistically significant (Highlighted in Bold).*

### Susceptibility Values of QSM Imaging between Groups

**FIGURE 3:**
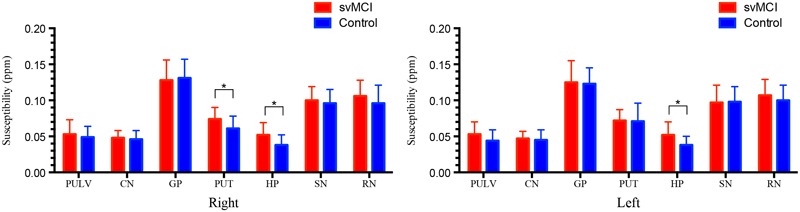
**Comparison of the susceptibility values in svMCI group and the controls.** Significant differences between svMCI group and controls are represented as ^∗^*p* < 0.05. svMCI, subcortical vascular mild cognitive impairment; PULV, pulvinar nucleus of the thalamus; CN, head of caudate nucleus; GP, globus pallidus; PUT, putamen; HP, hippocampus; SN, substantia nigra; RN, red nucleus.

The susceptibility values of the two groups are summarized in **Figure 3** and **Table 5**. The overall susceptibility value of the svMCI group was higher than that of control subjects, except for the right global pallidus. A significant difference in susceptibility values was found in bilateral hippocampus and right putamen in svMCI group compared with control group (right hippocampus: 0.052 ± 0.017, 0.038 ± 0.014, *p* < 0.01; left hippocampus: 0.052 ± 0.018, 0.038 ± 0.012, *p* < 0.01; right putamen: 0.074 ± 0.016, 0.061 ± 0.017, *p* < 0.05).

**Table 5:** Regional quantitative susceptibility mapping values for the svMCI group and control group.

		Susceptibility (ppm)		
		svMCI	Control	*p*-value
Pulvinar nucleus of the thalamus	Right	0.053 ± 0.020	0.049 ± 0.015	0.469
	Left	0.053 ± 0.017	0.044 ± 0.015	0.120
Head of caudate nucleus	Right	0.048 ± 0.010	0.046 ± 0.012	0.553
	Left	0.047 ± 0.010	0.045 ± 0.014	0.494
Globus pallidus	Right	0.128 ± 0.028	0.131 ± 0.026	0.788
	Left	0.125 ± 0.030	0.123 ± 0.022	0.783
Putamen	Right	0.074 ± 0.016	0.061 ± 0.017	**0.024**
	Left	0.072 ± 0.015	0.071 ± 0.025	0.805
Hippocampus	Right	0.052 ± 0.017	0.038 ± 0.014	**0.010**
	Left	0.052 ± 0.018	0.038 ± 0.012	**0.010**
Substantia nigra	Right	0.100 ± 0.019	0.096 ± 0.019	0.522
	Left	0.097 ± 0.024	0.098 ± 0.021	0.907
Red nucleus	Right	0.106 ± 0.022	0.096 ± 0.025	0.221
	Left	0.107 ± 0.022	0.100 ± 0.021	0.270

*svMCI, subcortical vascular mild cognitive impairment. Dates are given in means ± standard deviations; *p-*value < 0.05 was considered to be statistically significant (Highlighted in Bold).*

### Correlation between Regional Susceptibility Values and *Z*-Scores

**FIGURE 4:**
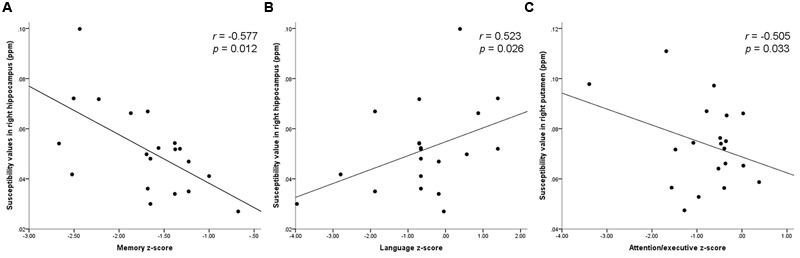
**Scatter plot illustrating the relationship between (A)** susceptibility values in right hippocampus and memory *z*-score, **(B)** susceptibility values in right hippocampus and language *z*-score, **(C)** susceptibility values in right putamen and attention-executive *z*-score.

In svMCI group, significantly negative correlations were observed between the susceptibility value of right hippocampus and memory *z*-score (*r* = -0.577, *p* = 0.012). The susceptibility value of the right hippocampus was positively correlated with the language *z*-score (*r* = 0.523, *p* = 0.026). The susceptibility value in the right putamen was negatively correlated with attention-executive *z*-score in the svMCI group (*r* = -0.505, *p* = 0.033). However, the composite *z*-score was not related to susceptibility values (shown in **Figure 4** and **Table 6**).

**Table 6:** Correlations of susceptibility values within bilateral hippocampus and right putamen in svMCI group.

		Right	Left	Right
		hippocampus	hippocampus	putamen
Composite	*p*-value	0.700	0.240	0.238
	*r*-value	0.098	-0.292	-0.293
Att-exe	*p*-value	0.821	0.564	**0.033**
	*r*-value	-0.057	-0.146	-**0.505**
Memory	*p*-value	**0.012**	0.630	0.925
	*r*-value	-**0.577**	-0.122	0.024
Language	*p*-value	**0.026**	0.626	0.994
	*r*-value	**0.523**	-0.123	0.002
Visuospatial	*p*-value	0.487	0.065	0.054
	*r*-value	-0.175	-0.443	-0.462

*svMCI, subcortical vascular mild cognitive impairment; Composite, composite *z*-score; Att-exe, attention-executive *z*-score; Memory, memory *z*-score; Language, language *z*-score; Visuospatial, visuospatial *z*-score. *p*-value < 0.05 was considered to be statistically significant (Highlighted in Bold).*

## Discussion

In the present study, the QSM technique was used to estimate the possible alterations in iron accumulation in the brains of svMCI subjects. Here, we observed that the svMCI group had higher iron concentrations in most subcortical nuclei than the control group. Iron was mainly deposited in the bilateral hippocampus and right putamen of the svMCI group. Furthermore, the susceptibility value in the right putamen and attention-executive *z*-score of the svMCI group were inversely related. We also found that the susceptibility value in the right hippocampus was negatively correlated with memory *z*-score and positively correlated with language *z*-score. However, composite *z*-score were not related to iron accumulation in the svMCI group. These observed group differences and clinical relevance of susceptibility value provide important implications for svMCI-related mechanisms, which could be detected by QSM.

Protein-associated iron is involved in many fundamental biological processes in the brain, such as oxidative phosphorylation, oxygen transportation, myelin production, and neurotransmitter synthesis and metabolism (Ward et al., 2014). However, excess iron may induce oxidative stress injury to propagate tissue damage and neurodegeneration (Rouault, 2001; Hametner et al., 2013). Neurodegeneration may result from iron-induced apoptosis and ferroptosis, an iron-specific form of non-apoptotic cell death (Ott et al., 2007; Dixon et al., 2012; Ward et al., 2014). Iron deposits and ferritin concentrations in the microglia and astrocytes of the cortex, basal ganglia, amygdala, hippocampus, and cerebellum generally increase with age. Iron deposition in specific brain regions is associated with motor and cognitive impairment (Ward et al., 2014). In our study, the age distributions of the svMCI and control groups were similar to eliminate age-associated effects. Changes in iron homoeostasis alter cellular iron distribution and accumulation in neurodegenerative diseases (Bartzokis et al., 2000; Collingwood and Dobson, 2006; Ward et al., 2014). Then, elevated iron levels may catalyze free-radical mediated damage to exacerbate the neurodegenerative stage (Filomeni et al., 2012). Traditionally, however, SVD is different from neurodegenerative diseases because it is induced by subcortical lesions and the incomplete infarction of white matter. We found that iron deposition in the hippocampus and putamen may be a biomarker of svMCI. Moreover, iron deposition in these regions has a similar accumulation pattern to those of AD or VD (Zhu et al., 2009; Raven et al., 2013; Moon et al., 2016). Therefore, these accumulation patterns occur not only in AD or VD, but also in svMCI patients. svMCI or other forms of SVD also contribute to increased iron accumulation in the deep brain nuclei and basal ganglia (Liem et al., 2012; Liu et al., 2015a).

Whether the iron accumulation noted in svMCI is a secondary effect or a primary event is not yet fully elucidated. Iron accumulation might be a result of svMCI. The atrophy of the cerebral cortex and the demyelination of white matter can decrease iron demand from storage areas in the deep gray nuclei, thus causing chronic iron accumulation (Dietrich and Bradley, 1988; Liem et al., 2012). Alternatively, iron deposition could also cause svMCI given that abnormal iron deposition causes white-matter disruption and atrophy (Ling et al., 2011). Iron is implicated in the pathogenesis of WMHs given that iron concentrations in the basal ganglia affect the severity of WMHs (Yan et al., 2013). However, other studies have suggested that iron accumulation is closely associated with the formation of cerebral microbleeds, but not with WMHs in SVD (Gattringer et al., 2016; Liu et al., 2016). Therefore, the precise mechanisms of higher iron concentration in svMCI still remain unclear and require further research.

We expect QSM to have a crucial quantitative role in establishing the mechanisms involved in brain iron changes in svMCI. QSM can accurately measure the susceptibilities of iron distribution in the deep brain nuclei and basal ganglia (Wang and Liu, 2015). Iron metabolism in normal aging, movement, and neurodegenerative disorders is an active area of research by QSM (Acosta-Cabronero et al., 2013; He et al., 2015; Darki et al., 2016; Moon et al., 2016). However, increased iron accumulation has rarely been demonstrated through QSM in svMCI or other form of SVD. Using high-resolution T2^∗^-weighted imaging, a study revealed increased diffuse iron accumulation in the putamen and caudate nucleus of patients with small-vessel disease cerebral autosomal dominant arteriopathy with subcortical infarcts and leukoencephalopathy (CADASIL). This result supported that SVD contributes to increased iron accumulation in the general population (Liem et al., 2012). Brain iron deposition also could be a biomarker of SVaD. It has been demonstrated by SWI that SVaD patients had abnormal iron deposition in widely cortical areas including hippocampus, which was related to neuropsychological scores (Liu et al., 2015a). Another study assessed iron deposition automatically following manual editing found iron accumulation might be an indicator of SVD that predispose to white matter damage which affecting the neuronal networks underlying higher cognitive functioning (Valdes Hernandez et al., 2016). In the present study, our QSM results showed similar patterns in svMCI. To our knowledge, the current results constitute the first attempt to estimate QSM alterations in the brains of svMCI patients. We found that the most striking deep gray matter feature is a marked increase in magnetic susceptibility in the putamen and hippocampus. Given that svMCI is a prodromal stage of SVaD, our finding may provide a potential biomarker for early diagnosis before clinical deterioration begins.

Our results indicated that the susceptibility value in the hippocampus was negatively correlated with memory *z*-score in the svMCI brain. Several neuroimaging studies have found hippocampal atrophy in svMCI or SVaD (Kim et al., 2014, 2015; Li et al., 2016). The hippocampus is susceptible to ischemia and lower blood volume; thus, hippocampal changes may be attributed to delayed neuronal death caused by chronic ischemia. An animal model of ischemia demonstrated that reducing cerebral blood flow causes memory and behavioral impairments and neuronal loss in the hippocampus (Roman et al., 2002). The hippocampus plays important roles in multiple memory systems (Schwarting and Busse, 2016). Given that the svMCI patients had a worse memory *z*-score— which reflects memory processing (i.e., auditory information retrieval processing, visuoperception, and short-term visual memory)— than the controls, we assume that the poor memory output of svMCI patients may be partially attributed to iron accumulation in the hippocampus. On the other hand, the susceptibility value of the hippocampus is positively correlated with the language *z*-score, which reflects language function. We found that svMCI patients exhibited more severe cognitive impairments in attention-executive and memory functions than in language function. However, the reason for the positive correlation between iron accumulation in the hippocampus and language *z*-score remains unclear. The speech-dominant hemisphere’s hippocampus plays a key role in language function, particularly naming. Naming function and functional MRI activation in the left hippocampus were significantly correlated (Bonelli et al., 2012). Thus, it is possible that altered iron distribution affects cognitive function. Further studies, however, are needed to clarify these issues. The susceptibility value in the putamen and attention-executive *z*-score were inversely related in svMCI subjects. Recent studies have shown that as part of the striatum, the putamen mainly regulates movements and influences various types of learning. The putamen is also involved in the emergence of dementia in neurodegenerative disorders, indicating its effect on cognitive impairment (de Jong et al., 2008). Putamenal lesions might cause behavioral and cognitive changes. Putamenal hemorrhages could disrupt dorsolateral-striato-pallido-thalamic circuits and cause executive dysfunction (Kokubo et al., 2015). The putamen also plays an important role in basal ganglia-thalamic circuits that are involved in attentive processing (Tortorella et al., 2013). The present study is in line with these results. Hence, our findings may further confirm the role of the putamen in cognitive function, especially in attention-executive function. Alterations in putamen susceptibility values and its relationships with the *z*-score suggest that putamen could be linked to the pathophysiology of svMCI and indicate the clinical relevance as a biomarker.

This study has several limitations. First, we did not include a control group of healthy elderly subjects. Second, the MRI and neuropsychological test were not performed simultaneously. Third, the relatively small number of patients limited our cross-sectional study. Thus, a longitudinal study that follows large cohorts of svMCI patients throughout their conversion to SVaD is crucial to investigate the dynamic course of iron deposition and confirm the physiopathological processes of SVaD. Fourth, as with all *in vivo* MRI studies of svMCI, our study was limited by the lack of pathologically confirmed patients, although we diagnosed the patients with both comprehensive neuropsychological assessments and MRI. Finally, the precise mechanisms that led to higher iron concentration in svMCI patients still remain unclear and should be further investigated.

## Conclusion

We found that QSM is a feasible technique for measuring iron deposition in the subcortical nuclei. Iron was mainly deposited in the bilateral hippocampus and right putamen of the svMCI group. The relationship between the susceptibility value in the putamen and attention-executive *z*-score, and between the susceptibility value in the hippocampus and memory *z*-score, implicate the putamen and hippocampus in the pathophysiology of svMCI. Furthermore, these relationships could explain the cognitive disturbances seen in the svMCI group. Our results indicated that the association between increased brain iron-burden and neurocognitive dysfunction was caused by svMCI. Moreover, our results provide evidence that accumulated subcortical iron is a biomarker for the pathophysiological mechanism of neural and cognitive decline in the evolution of svMCI.

## Author Contributions

Conceived and designed the experiments: YaZ, QX, and JX. Performed the experiments: XG, XH, WC, YW, WD, and MC. Analyzed the data: YS and WC. Contributed reagents/materials/analysis tools: WC and YZ. Wrote the paper: YS and XG. Figures processing: YS.

## Conflict of Interest Statement

The authors declare that the research was conducted in the absence of any commercial or financial relationships that could be construed as a potential conflict of interest.
